# [^177^Lu]Lu-DOTA-ZOL bone pain palliation in patients with skeletal metastases from various cancers: efficacy and safety results

**DOI:** 10.1186/s13550-020-00709-y

**Published:** 2020-10-28

**Authors:** Madhav Prasad Yadav, Sanjana Ballal, Marian Meckel, Frank Roesch, Chandrasekhar Bal

**Affiliations:** 1 Department of Nuclear Medicine, Room No: 59-A, Thyroid Clinic, All India Institute of Medical Sciences (AIIMS), Ansari Nagar, New Delhi, 110029 India; 2 Department of Nuclear Chemistry, Johannes Gutenberg University, Fritz-Strassmann-Weg 2, 55126 Mainz, Germany

**Keywords:** [^177^Lu]Lu-DOTA-ZOL, Pain palliation, Skeletal metastases

## Abstract

**Background:**

[^177^Lu]Lu-DOTA-ZOL has shown promising results from the dosimetry and preclinical aspects, but data on its role in the clinical efficacy are limited. The objective of this study is to evaluate the efficacy and safety of [^177^Lu]Lu-DOTA-ZOL as a bone pain palliation agent in patients experiencing pain due to skeletal metastases from various cancers.

**Methods:**

In total, 40 patients experiencing bone pain due to skeletal metastases were enrolled in this study. The patients were treated with a mean cumulative dose of 2.1 ± 0.6 GBq (1.3–2.7 GBq) [^177^Lu]Lu-DOTA-ZOL in a median follow-up duration of 10 months (IQR 8–14 months). The primary outcome endpoint was response assessment according to the visual analogue score (VAS). Secondary endpoints included analgesic score (AS), global pain assessment score, Eastern Cooperative Oncology Group Assessment performance status (ECOG), Karnofsky performance status, overall survival, and safety assessment by the National Cancer Institute’s Common Toxicity Criteria V5.0.

**Results:**

In total, 40 patients (15 males and 25 females) with a mean age of 46.6 ± 15.08 years (range 24–78 years) were treated with either 1 (*N* = 15) or 2 (*N* = 25) cycles of [^177^Lu]Lu-DOTA-ZOL. According to the VAS response assessment criteria, complete, partial, and minimal responses were observed in 11 (27.5%), 20 (50%), and 5 patients (12.5%), respectively with an overall response rate of 90%. Global pain assessment criteria revealed complete, partial, minimal, and no response in 2 (5%), 25 (62.5%), 9 (22.5%), and 4 (10%) patients, respectively. Twenty-eight patients died and the estimated median overall survival was 13 months (95% CI 10–14 months). A significant improvement was observed in the VAS, AS, and ECOG status when compared to baseline. None of the patients experienced grade III/IV haematological, kidney, or hepatotoxicity due to [^177^Lu]Lu-DOTA-ZOL therapy.

**Conclusion:**

[^177^Lu]Lu-DOTA-ZOL shows promising results and is an effective radiopharmaceutical in the treatment of bone pain due to skeletal metastases from various cancers.

## Introduction

Bone is the most common site of metastases in the majority of the solid cancers. Skeletal metastases from prostate and breast cancer account for approximately 80% of all the bone metastases followed by lung and renal cancers that comprise 20–40% of all the patients [1]. The typical clinical symptom of skeletal metastases is bone pain. Apart from pain, other skeletal-related events (SREs), albeit to a lesser extent, are swelling, nerve compression, immobility, or pathological fractures. At the same time, few patients are even asymptomatic, particularly in the early part of the disease with marrow metastases.

Several algorithms have been evolved over the last 3 decades for the management of metastatic bone pain and SREs [2] in which a range of systemic to locoregional therapies are advocated. The most common approaches in clinical practice are chemotherapy, hormonal therapy, bisphosphonates, monoclonal antibody, namely denosumab and analgesics like non-steroidal anti-inflammatory drugs and opioids, molecules signalling growth factors, antidepressants, and endothelin receptor antagonists. Locoregional therapies are offered only for patients with oligo-metastases, namely, external beam radiotherapy (EBRT). Even though the list seems vast, none of them are curative in practice, with a majority of patients limited to palliative care, and involve a multidisciplinary approach.

Palliative external beam radiotherapy is the most effective way of bone pain management. Studies have revealed that 80–90% of patients receiving EBRT have demonstrated complete or partial pain relief within 10–14 days from initiation of treatment. However, EBRT, as mentioned above, is only limited to the treatment of oligo-metastases or in a worst-case scenario to hemi-body irradiation [3].

Radionuclide therapy is a systemic form of internal radiotherapy which constitutes an essential option as a routine part of the multidisciplinary treatment approach for decades. Several beta-emitting radiometals have been exploited for this purpose that includes ^89^Sr ^186^Re, ^188^Re, and ^153^Sm. All of them have been incorporated into bone-seeking phosphonates, except ^89^Sr. The ^89^Sr is a bivalent cation sharing properties similar to calcium. The same is true for ^223^Ra, the most recent radionuclide which has been approved for pain palliation in prostate cancer without visceral metastasis [4], except that it is an alpha emitter. However, the availability and the cost of ^223^Ra pose paramount restraints for most of the developing world. In this scenario, beta emitters are still affordable and an acceptable option. Owing to the suitable physical properties and decay characteristics of ^177^Lu [*t*1/2 = 6.73 days Eβmax = 497 keV, Eγ) = 113 keV (6.4%), 208 keV (11%)], it is now widely used in clinical practice. It is either produced via the ^176^Lu(n,γ) [5] or the ^176^Yb(n,γ) pathway [6].

For bone pain palliation it has been labelled with several bisphosphonates such as ethylene diamine tetramethylene phosphonic acid (EDTMP) [7–9], (4-{[(bis(phosphonomethyl)) carbamoyl]methyl}-7,10-bi (carboxymethyl)-1,4,7,10-tetraazacyclododec-1-yl) acetic acid (BPAMD) [10–15] Zoledronate (DOTA-ZOL) [16–22], and DOTMP [23, 24]. Zoledronic acid, compared to EDTMP and DOTMP, has significantly higher hydroxyapatite binding and better internalisation by osteoclasts. These superior antiresorptive properties lead to increased apoptosis [25]. DOTA-ZOL can be labelled with both ^68^Ga and ^177^Lu to form a theranostic pair [19]. However, only two studies have reported its safety from the dosimetric aspects [26, 27]. Recently, in a pilot study, Nikzad et al. [26] labelled DOTA-zoledronate with ^177^Lu and have shown promising results and comparable pharmacokinetics to [^177^Lu]Lu-EDTMP. While dosimetry data revealed a higher absorbed dose for [^177^Lu]Lu-DOTA-ZOL compared to [^177^Lu]Lu-EDTMP (12.17 vs. 10.02 mSv/MBq) in the trabecular bone surface, the absorbed dose to the critical organs and the muscle from [^177^Lu]Lu-DOTA-ZOL was much lower compared to that of [^177^Lu]Lu-EDTMP [27] consistent with the results of the above study. Khawar et al. [21] revealed a similar biodistribution and normal organ absorbed doses of [^177^Lu]Lu-DOTAZOL. However, to the best of our knowledge, investigation on the efficacy and safety of [^177^Lu]Lu-DOTA-ZOL for pain palliation in a clinical setting has not been reported to date. Hence, in the present study, we aim to report the efficacy and toxicity data of the patients, treated with [^177^Lu]Lu-DOTA-ZOL, for the bone pain palliation of skeletal metastases from various cancers.

## Materials and methods

The study was conducted at the Department of Nuclear Medicine, AIIMS, New Delhi, India. Skeletal metastases patients suffering from pain were referred from the Pain Clinic, Medical Oncology, and Radiation Oncology departments for [^177^Lu]Lu-DOTA-ZOL pain palliation treatment. This cohort study involved patients who were treated with [^177^Lu]Lu-DOTA-ZOL for pain palliation between January 2017 and February 2020.

### Eligibility criteria

Eligibility criteria for the [^177^Lu]Lu-DOTA-ZOL pain palliation treatment included: histologically confirmed breast, prostate, or lung cancers, progressive pain or pain requiring escalation of analgesics, patients with more than one site of pain corresponding to the avid uptake on [^68^Ga]Ga-DOTA-ZOL PET/CT scan, patients with no prior history of radionuclide pain palliation therapy, Eastern Cooperative Oncology Group (ECOG) performance status ≤ 4, KPS ≥ 50, patient on or with history of prior bisphosphonates, patients with haematological, kidney, and liver function parameters within normal limits which included baseline haemoglobin of < 9 g/dL, platelet counts: < 75,000/μL, leukocyte counts: ≥ 4 × 10^9^/L, serum creatinine: > 1.4 mg/dL, serum bilirubin > 3 mg%, glomerular filtration rate (GFR): < 50 mL/min per 1.73 m^2^ body surface area (BSA). Patients with skeletal-related events involving pathological fractures and cord compression were not included.

### [^177^Lu]Lu-DOTA-ZOL synthesis

The stock solution consisted of 1 mg DOTA-ZOL dissolved in 1 mL ultrapure water to give a concentration of 60 µg/60 µL. The 60 µL of DOTA-ZOL was radiolabelled with [^177^Lu]LuCl_3_ which was obtained from BRIT, India, in sodium ascorbate buffer, pH 4, in 0.01 M supra pure HCl with a specific activity ranging between 370 and 740 GBq/mg. The radiolabelled solution was heated at 95 °C for 30 min. Radiochemical quality control was carried out using the instant thin-layer chromatography method with sodium citrate buffer as the solvent.

### Treatment protocol and follow-up

#### [^177^Lu]Lu-DOTA-ZOL infusion

**Fig. 1:**
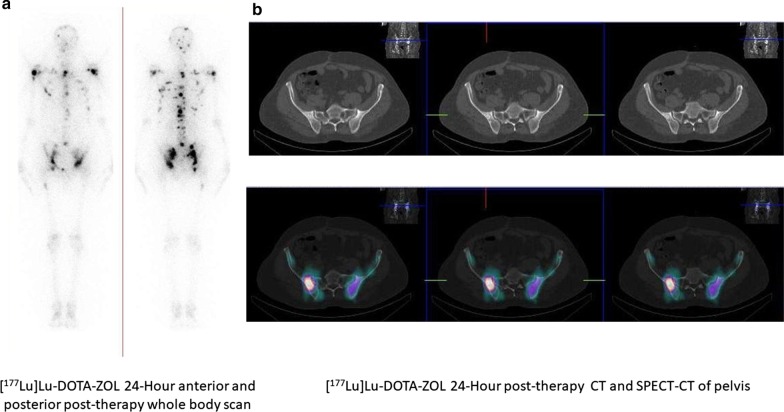
A 55-year-old male diagnosed with prostatic adenocarcinoma underwent radical prostatectomy in April 2017, and his Gleason score was 3 + 4 = 7. He underwent hormonal therapy until April 2018 and received 1# of radiotherapy to B/L hip and D5-vertebra. He was referred to us for symptomatic pain relief. His baseline pain scoring (VASmax) was 9/10, with maximum pain in the B/L hips. We administered 1.3 GBq of [^177^Lu]Lu-DOTA-ZOL and 24-h post-administration, **a** the 24-h anterior and posterior post-therapy whole-body scans demonstrating avid [^177^Lu]Lu-DOTA-ZOL uptake in multiple skeletal sites. **b** The uptake of [^177^Lu]Lu-DOTA-ZOL in the pelvic bone metastases on post-therapy SPECT/CT

**Fig. 2:**
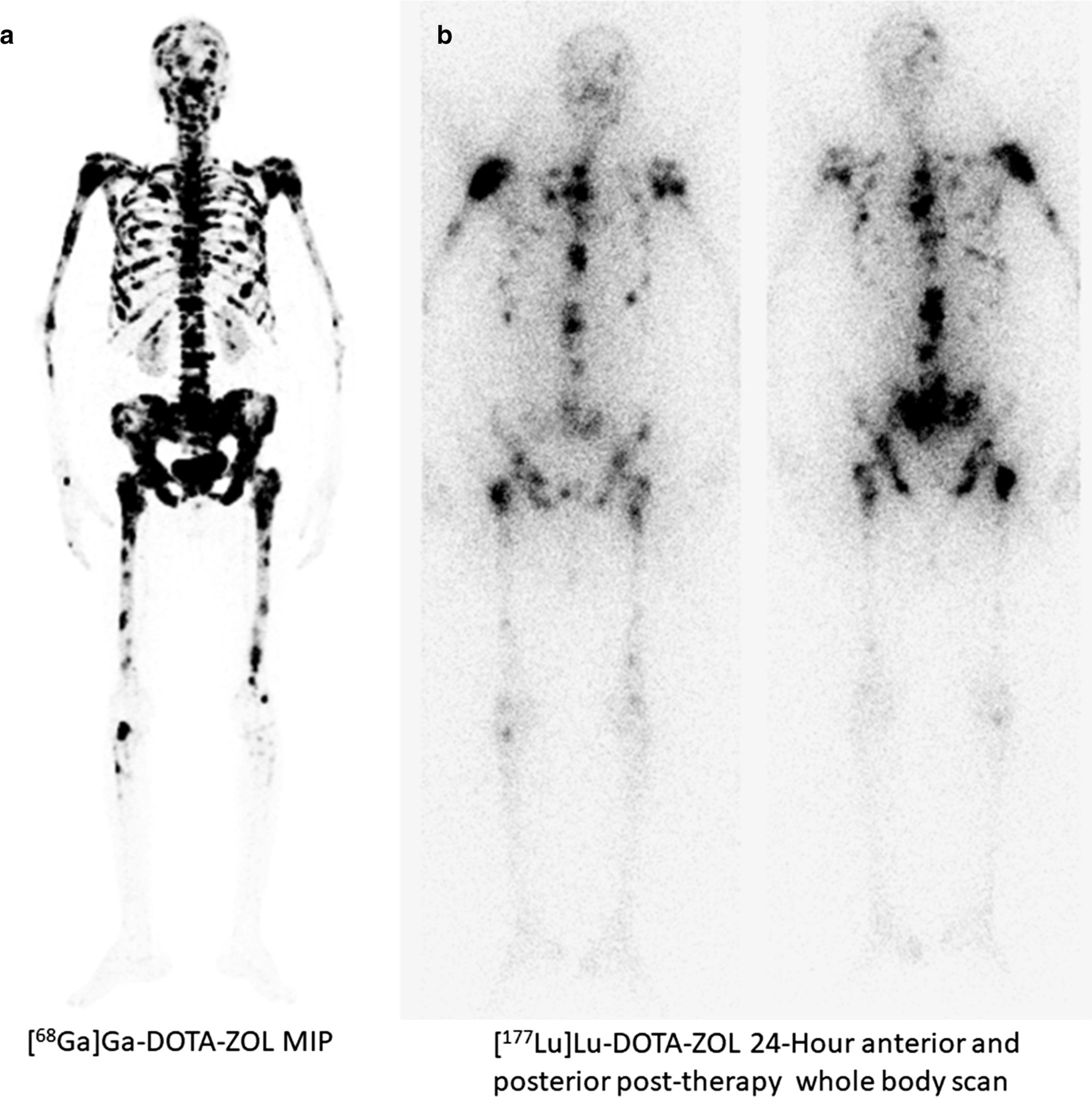
A 45-year-old female, diagnosed with triple-negative breast cancer was on opioid medications, but did not experience any relief in pain. Her baseline pain scoring (VASmax) was 8/10. Baseline [^68^Ga]Ga-DOTA-ZOL PET/CT (**a**) was conducted followed by administration of 1.3 GBq of [^177^Lu]Lu-DOTA-ZOL. Twenty-four-hour post-administration, post-therapy 24-h anterior and posterior whole body scan (**b**) demonstrate avid [^177^Lu]Lu-DOTA-ZOL uptake in multiple skeletal sites

The patients who fulfilled the eligibility criteria were administered with a fixed dose of 1295 MBq (35 mCi) [^177^Lu]Lu-DOTA-ZOL. The fixed activity of 1295 MBq of [^177^Lu]Lu-DOTA-ZOL was extrapolated from our previous [^177^Lu]Lu-EDTMP phase II data [8]. The infusion involved a dilution of [^177^Lu]Lu-DOTA-ZOL in 10 mL normal saline (0.9%), which was administered intravenously over 5 min, with subsequent flushing of 20 mL normal saline. The entire process was performed on an in-patient basis, and patients were discharged in a few hours of observation if they do not show any adverse reaction to [^177^Lu]Lu-DOTA-ZOL. Figure 1 shows biodistribution and uptake of [^177^Lu]Lu-DOTA-ZOL therapy in a patient with skeletal metastases from prostate cancer, while Fig. 2 gives similar data of [^177^Lu]Lu-DOTA-ZOL therapy in a patient with skeletal metastases from breast cancer.

#### Follow-up

The treatment was repeated if necessary at 3-monthly intervals. Post-[^177^Lu]Lu-DOTA-ZOL administration, patients were assessed at 2, 4, 8, and 12 weeks. Patients were assessed for laboratory parameters, and the adverse events were recorded according to the National Cancer Institute for Common Toxicity Criteria version (CTC) 5.0 [28]. The visual analogue score (VAS) [29], global pain assessment score, analgesic score (AS), Karnofsky performance status (KPS) [30, 31], and assessment of pain relief were recorded in the patient case files on each visit. Patients were instructed to maintain a diary and document the pain relief parameters such as initiation of pain relief, duration of pain relief, time of increase and/or recurrence of pain, decrease or increase in the consumption of pain killers.

### Treatment response assessment

#### Primary outcome endpoint

The primary outcome endpoint was response assessment by VAS. According to this criteria, the complete response (CR), partial response (PR), minimal response(MR), and no response (NR) were categorised as > 70% reduction, 40–70% reduction, 20–40% reduction, and < 20% decrease in VAS or increase in pain, respectively [29].

#### Secondary outcome endpoints

Other clinical response assessment parameters involved analgesic score (AS), Karnofsky performance status (KPS), Eastern Cooperative Oncology Group (ECOG) performance status, global pain assessment, adverse event profile, and overall survival.

Analgesic scoring was conducted as per the Urological Group of the European Organization of Research and Treatment of Cancer (EORTC, Protocol 30921). As per EORTC protocol, the analgesic score is the product of two five-point scales (the type of analgesic and the frequency of its administration). A decline in the analgesic score was documented as a response to treatment.

Additionally, global pain response assessment was analysed according to the criteria adopted by Thapa et al. [32] that considered changes in both VAS and analgesic scores (rather than a single parameter). According to the criteria, the present study design considered post-therapy changes in both VAS and analgesic scores on a sliding scale. The global pain assessment criteria are accordingly complete (75% decrease in analgesic score with change in pain score), partial (50–75% decrease in analgesic score with a change in pain score), minimal (25–50% decrease in analgesic score with a change in pain score), or none (no change in pain score or, 25% decrease in the analgesic score). The KPS was scaled from 100 to 0. The ECOG status ranged from 0 to 5. All adverse events were assessed as per the National Cancer Institute’s Common Toxicity Criteria (NCI-CTC) version 5.0. The overall survival (OS) was defined as the time from the initiation of [^177^Lu]Lu-DOTA-ZOL treatment to the time of death. The death could be attributed to any cause or the last telephonic contact.

### Statistical analysis

The normality of the data was examined by the D’Agostino–Pearson test. The data were presented as mean standard deviation (SD), median, and/or interquartile range (IQR). Unpaired samples *t* test (parametric test) or Mann–Whitney *U* test (nonparametric test) was performed for two independent patient groups. The paired *t* test (parametric test) or Wilcoxon signed-rank test (nonparametric test) was executed to compare parameters at pre- and post-treatment time points. Kaplan–Meier curves analysis was conducted to calculate the overall survival. MedCalc software was used for statistical analyses. *P* values ≤ 0.05 were considered significant.

## Results

### Patients

Forty documented skeletal metastases patients including 15 males and 25 females with a mean age of 46.6 ± 15.08 years (range 24–78 years) were enrolled and treated with [^177^Lu]Lu-DOTA-ZOL for bone pain palliation therapy. The patients were treated between January 2017 and February 2020 with the median follow-up duration of 10 (IQR 8–14) months.

The baseline demographic profile of the patients, tumour characteristics, previous and ongoing cancer-related treatments, and the analgesics consumed are outlined in Tables 1 and 2. Among the patients treated, breast cancer (23/40, 57.5%) accounted for the maximum number of cases followed by prostate cancer (11/40, 27.5%). The remaining 6 patients had lung cancer (Table 1). Except eight patients with prostate cancer who were on concomitant hormonal therapy, no other patients were on any anti-cancer treatment during the treatment. While 15 (37.5%) patients were on morphine medications at the baseline, the remaining patients (62.5%) were either on atypical opioids, non-morphine opioids, or other NSAIDs. Before being referred to our department for bone pain palliation, all the patients had undergone a minimum of two lines of prior treatment.

**Table 1:** Patient clinical characteristics

Variables	Number (%)
Age in years (mean ± SD, range)	46.6 ± 15.08 (24–78)
Gender	
Male	15 (37.5%)
Female	25 (62.5%)
Primary disease	
Prostate	11 (27.5%)
Breast	23 (57.5%)
Lung	6 (15%)
Baseline VAS pre-therapy (median, IQR)	9 (8–10)
Baseline KPS pre-therapy (median, IQR)	60 (50–70)
Baseline analgesic score (median)	6 (6–8)
The extent of skeletal metastases	
< 6	2 (5%)
6–20	17 (42.5%)
> 20	7 (17.5%)
Diffuse/superscan	14 (35%)
ECOG performance status	
2	12 (30%)
3	15 (37.5%)
4	13 (32.5%)
Mean cumulative activity (GBq)	2.1 ± 0.6 GBq (1.3–2.7 GBq)

*IQR* inter-quartile range, *VAS* visual analogue score, *KPS* Karnofsky performance status, *ECOG* Eastern Cooperative Oncology Group, *GBq* gigabecquerel

**Table 2:** Detailed clinical history of patients

Patient	Age/gender	Type of cancer	Previous therapy	Ongoing treatment	Analgesic score (type)	Analgesic score (quantity)
1	51/M	mCRPC	First- and second-generation anti-androgens, chemotherapy	Androgen synthesis inhibitor	Morphine	2
2	25/F	Non-small cell right lung cancer	Lobectomy, chemotherapy, radiotherapy	–	Morphine	2
3	60/M	Right breast cancer	Chemotherapy, radiotherapy	–	Morphine	2
4	44/F	B/L breast cancer	B/L breast mastectomy, chemotherapy, radiotherapy, monoclonal antibody therapy	–	Morphine	2
5	61/M	Right breast cancer	Hormonal therapy, chemotherapy, radiotherapy	–	Atypical opioids and non-morphine opioids	3
6	70/M	mCRPC	Medical castration, first-generation anti-androgen therapy, chemotherapy, radiotherapy	Second-generation anti-androgens	Morphine	4
7	63/M	mCRPC	B/L orchidectomy, first-generation anti-androgens, chemotherapy, radiotherapy	–	Atypical opioids and non-morphine opioids	2
8	60/F	Small-cell lung cancer	Chemotherapy, radiotherapy	–	Atypical opioids and non-morphine opioids	3
9	44/F	Right breast cancer	Right breast mastectomy, hormonal therapy, chemotherapy, radiotherapy	–	Atypical opioids and non-morphine opioids	2
10	65/F	Left breast cancer	Hormonal therapy, chemotherapy,	–	Atypical opioids and non-morphine opioids	2
11	33/F	Right breast cancer	Chemotherapy, radiotherapy	–	Morphine	2
12	74/M	mCRPC	Radical Prostatectomy, B/L orchidectomy, first-generation anti-androgens, chemotherapy,	-	Atypical opioids and non-morphine opioids	2
13	45/F	Left breast cancer	Left breast modified radical mastectomy, hormonal therapy, chemotherapy, radiotherapy,	–	Atypical opioids and non-morphine opioids	3
14	27/F	B/L breast cancer	Hormonal therapy, B/L mastectomy, chemotherapy, radiotherapy,	–	Morphine	2
15	47/F	Left breast cancer	Hormonal therapy, chemotherapy, radiotherapy, antibody therapy	–	Other NSAIDs	1
16	27/F	Left breast cancer	chemotherapy, radiotherapy,	–	Other NSAIDs	2
17	55/M	mCRPC	B/L orchidectomy, first- and second-generation anti-androgens, chemotherapy, radiotherapy	Androgen synthesis inhibitors	Other NSAIDs	3
18	44/F	Left breast cancer	Left modified radical mastectomy, chemotherapy, radiotherapy	–	Morphine	2
19	58/F	Right breast cancer	Rt breast mastectomy chemotherapy, radiotherapy	–	Atypical opioids and non-morphine opioids	4
20	24/M	Squamous cell cancer of lung	Surgery, chemotherapy	–	Morphine	2
21	55/F	Left breast cancer	Surgery, chemotherapy, radiotherapy	–	Atypical opioids and non-morphine opioids	2
22	55/F	Squamous cell cancer of lung	Chemotherapy, radiotherapy	–	Atypical opioids and non-morphine opioids	2
23	54/F	Right breast cancer	Chemotherapy, radiotherapy	–	Atypical opioids and non-morphine opioids	3
24	40/F	Right breast cancer	Chemotherapy, radiotherapy	–	Morphine	2
25	54/M	mCRPC	Medical castration, Chemotherapy, radiotherapy	Androgen-synthesis inhibitors	Morphine	2
26	63/F	B/L breast cancer	Bilateral mastectomy, chemotherapy, radiotherapy	–	Atypical opioids and non-morphine opioids	2
27	47/F	Right breast cancer	Right breast mastectomy, hormonal therapy, chemotherapy, radiotherapy	–	Morphine	2
28	50/M	Right breast cancer	Right modified radical mastectomy, chemotherapy, radiotherapy	–	Morphine	2
29	40/F	B/L breast cancer	Hormonal therapy, chemotherapy, radiotherapy	–	Morphine	2
30	75/M	mCRPC	B/L orchidectomy, chemotherapy, first- and second-generation anti-androgens, radiotherapy	None	Atypical opioids and non-morphine opioids	2
31	67/M	mCRPC	B/L orchidectomy, first-generation anti-androgens Chemotherapy, radiotherapy,	Second-generation anti-androgens	Atypical opioids and non-morphine opioids	2
32	78/M	mCRPC	B/L orchidectomy, chemotherapy	Androgen synthesis inhibitor	Atypical opioids and non-morphine opioids	2
33	48/F	B/L breast cancer	Chemotherapy, radiotherapy	–	Atypical opioids and non-morphine opioids	2
34	40	Right breast cancer	Right modified radical mastectomy, chemotherapy, radiotherapy	–	Atypical opioids and non-morphine opioids	2
35	70/M	mCRPC	Medical castration, chemotherapy, radiotherapy	Androgen-synthesis inhibitors	Morphine	2
36	43/M	Non-small cell lung cancer of the right lung	Chemotherapy, radiotherapy	–	Atypical opioids and non-morphine opioids	2
37	32/F	Right breast cancer	Right breast mastectomy chemotherapy, radiotherapy	–	Atypical opioids and non-morphine opioids	2
38	67/F	Right breast cancer	Right modified radical mastectomy, hormonal therapy chemotherapy, radiotherapy	–	Atypical opioids and non-morphine opioids	2
39	30/F	Small cell cancer of lung	Chemotherapy, radiotherapy	–	Atypical opioids and non-morphine opioids	3
40	76/M	mCRPC	B/L orchidectomy, first-generation anti-androgens, chemotherapy, radiotherapy	Second-generation anti-androgens	Atypical opioids and non-morphine opioids	2

*B/L* bilateral, *mCRPC* metastatic castration-resistant prostate cancer

### Treatment cycles and efficacy assessment

The cumulative activity administered was 2.1 ± 0.6 GBq (range 1.3–2.7 GBq) (56.8 ± 17 mCi; range 35–70 mCi). A total of 65 cycles of [^177^Lu]Lu-DOTA-ZOL were administered in 40 patients, among whom, 25 patients received two cycles each and the remaining 15 patients received only 1 cycle. Flair phenomenon was noted in 7/40 (17.5%) of patients within 2–3 days of [^177^Lu]Lu-DOTA-ZOL treatment. These patients complained of persistent pain even on strong analgesics; however, it was transient and reduced in 7–10-day post-treatment. According to the VAS response criteria, complete response was demonstrated in 27.5% (11/40), partial response in 50% (20/40), the minimal response in 12.5% (5/40), and no response in 10% (4/40) of patients with an overall response rate (ORR) of 90%. Similarly, as per the global pain assessment response criteria, pain relief with [^177^Lu]Lu-DOTA-ZOL was 90%: 5% (2/40) CR, 62.5% (25/40) PR, and 22.5% (9/40) MR, respectively. The detailed classification of relation between, the extent of skeletal metastases, number of cycles administered, and responses according to both VAS criteria and global pain assessment criteria are explained in Table 3. Interestingly, Among 14 patients with superscan or diffuse involvement of skeletal metastases, only one patient did not respond to treatment. A similar pattern of responses was observed in patients with > 20 skeletal metastases (Table 3). Nine patients who attained minimal response after the first cycle were re-challenged with the second cycle of [^177^Lu]Lu-DOTA-ZOL, but found very minimal improvement despite the second cycle and remained in the minimal response category. Among the 15 patients who received only one cycle of [^177^Lu]Lu-DOTA-ZOL treatment, according to VAS criteria, 4 did not respond to treatment, 3 experienced only minimal response, and 8 patients responded well but did not consent for the 2nd cycle of treatment (Table 3).

**Table 3:** Response and number of [^177^Lu]Lu-DOTA-ZOL treatment cycles administered according to the extent of disease

Extent of skeletal metastases	Number of patients (%)	Response according to VAS assessment criteria (number of patients)	Number of cycles administered	Response according to global pain assessment criteria (number of patients)	Number of cycles administered
< 6	2 (5%)	CR-1	2C	PR-2	1st patient-1C
		PR-1	1C		2nd patient-2C
6–20	17 (42.5%)	CR-5	4 patients-2C, 1 patient-1C	CR-1	2C
		PR-9	5 patients-2C, 4 patients-1C	PR-11	8 patients-2C, 3 patients-1C
		MR-1	2C	MR-3	2 patients-1C, 1 patient-2C
		NR-2	Both patients-1C	NR-2	Both patients-1C
> 20	7 (17.5%)	CR-2	2 patients, 2C	CR-1	2C
		PR-4	3 patients-2C, 1 patient-1C	PR-4	3 patients-2C, 1 patient-1C
		MR-0	0	MR-1	2C
		NR-1	1C	NR-1	1C
Superscan/diffuse	14 (35%)	CR-3	All patients-2C	CR-0	–
		PR-6	5 patients-2C, 1 patient-1C	PR-8	6 patients-2C, 2 patients-1C
		MR-4	1 patient-2C, 3 patients-1C	MR-5	3 patients-2C, 2 patients-1C
		NR-1	1 patient 1C	NR-1	1C

*VAS* visual analogue score, *CR* complete response, *PR* partial response, *MR* minimal response, *NR* no response, *C* cycles

In the post-treatment period, there was a significant decrease in VAS [(pre-therapy: 9 (IQR 8–10) vs. post-therapy: 4 (IQR 3–5), *P* < 0.0001). Similarly, a remarkable improvement noted in the KPS [(pre-therapy: 60 (IQR 50–70) vs. post-therapy: 80 (IQR 60–80), *P* < 0.0001), and the ECOG performance status [(pre-therapy: 3 (IQR 2–4) vs. post-therapy: 2 (IQR 2–3), *P* 0.0013) post-[^177^Lu]Lu-DOTA-ZOL therapy. While there was a significant reduction in the analgesic score pre- and post-treatment with [^177^Lu]Lu-DOTA-ZOL in the CR, PR, and the MR categories, the same was not found in the NR category (Table 4).

**Table 4:** Comparison of pre- and post-treatment analgesic scores stratified according to VAS response criteria

Response (VAS criteria)	Number of patients (*N*)	Baseline AS (median, IQR)	Post-treatment AS (median, IQR)	*P* value
CR	11	6 (6–8)	2 (1.2–2.7)	0.001
PR	20	7 (6–8)	3 (3–3.5)	< 0.0001
MR	5	8 (6–8.25)	4.8 (4–6)	0.006
NR	4	8.5 (8–12.5)	8 (8–12)	0.918

All the values are mentioned as median and inter-quartile range (IQR)

*VAS* visual analogue score, *N* number of patients, *IQR* interquartile range, *CR* complete response, *PR* partial response, *MR* minimal response, *NR* no response

Response according to the type of cancer revealed 100% (23/23), 82% (9/11), and 66.6% (4/6) ORR in patients with breast, prostate, and lung cancer, respectively (Table 5).

**Table 5:** Response assessment according to the type of cancer

Response criteria	Breast cancer *N* = 23	Prostate cancer *N* = 11	Lung cancer *N* = 6
CR	9	2	0
PR	11	7	2
MR	3	0	2
NR	0	2	2

*N* number of patients, *CR* complete response, *PR* partial response, *MR* minimal response, *NR* no response

**Fig. 3:**
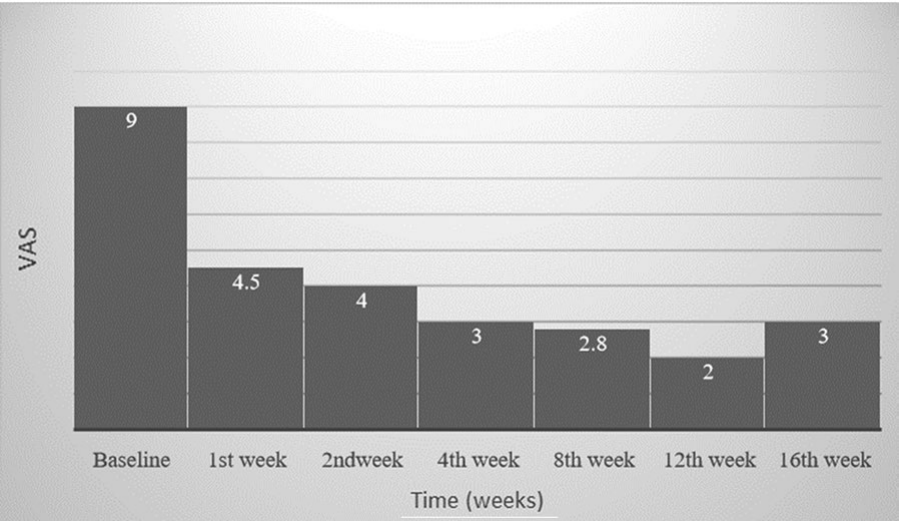
Plot showing the temporal relationship of the median value of visual analogue score (VAS) of patients from baseline up to 16 weeks of post-[^177^Lu]Lu-DOTA-ZOL therapy

The median time for the initiation of pain relief was ≤ 7 days (IQR 6–9 days) (Fig. 3). The median time of sustained response after the last cycle of the [^177^Lu]Lu-DOTA-ZOL is 3 months (IQR 2–4 months). Only one patient observed the most prolonged duration of sustained response, which was 10 months.

### Survival analysis

**Fig. 4:**
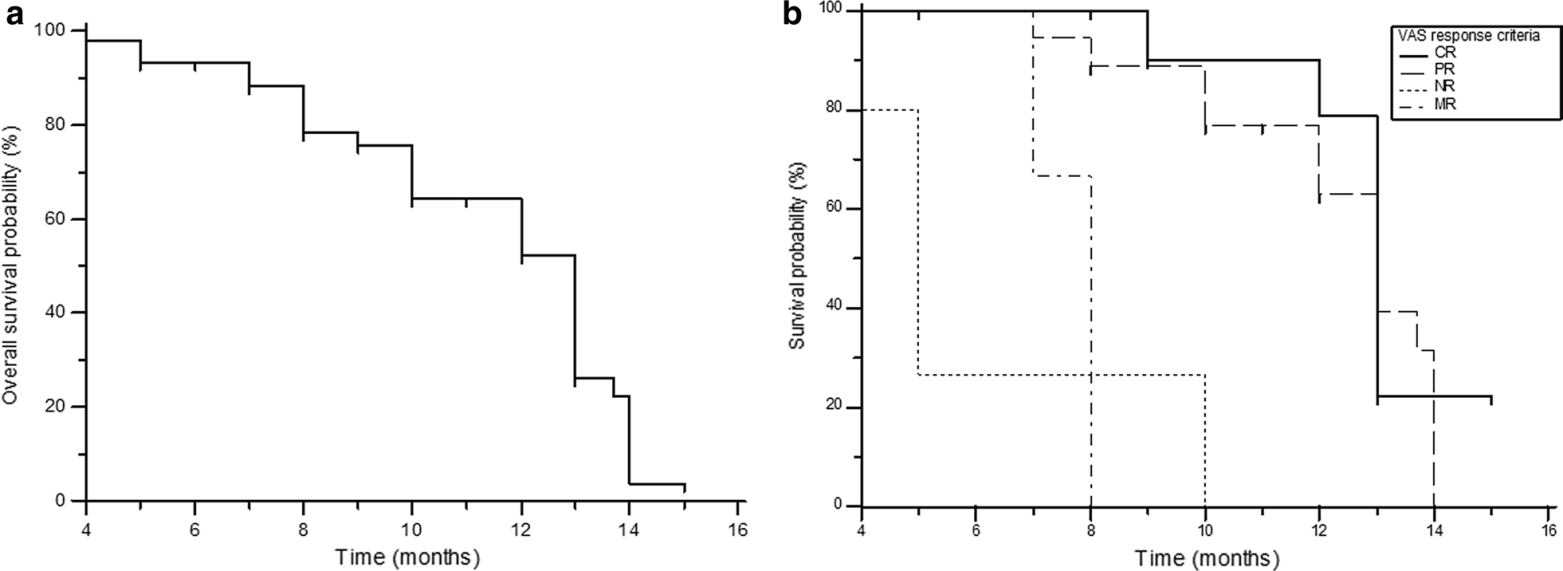
**a** Kaplan–Meier analysis: overall survival. **b** Overall survival, according to the response category as per VAS response criteria

**Fig. 5:**
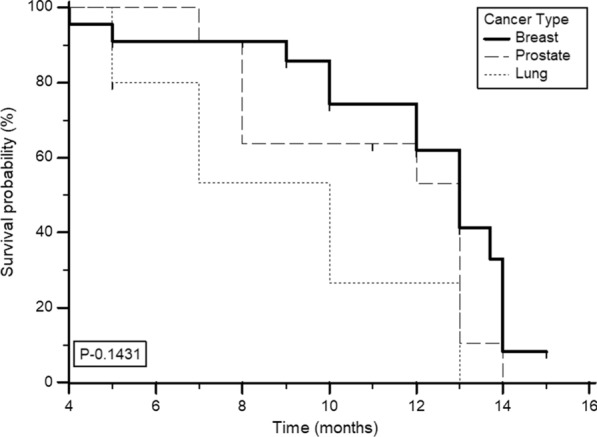
Kaplan–Meier analysis: overall survival according to the type of cancer

Among the entire series, 28 patients died during the follow-up. The median survival from treatment was 13 months (95% CI 10–14 months), with a 1-year survival probability of 55.4% (Fig. 4a). On subgroup analysis, in patients with CR, PR, MR, and NR, the median overall survival was 13, 13, 8, and 5 months, respectively (Fig. 4b). Sub-categorical analysis based on the type of cancer revealed patients with breast and prostate cancer to depict a similar median overall survival duration of 13 months, while patients with lung cancer demonstrated a median overall survival of 10 months. However, the log-rank test did not prove significant (*P* 0.1431) (Fig. 5).

### Toxicity

**Fig. 6:**
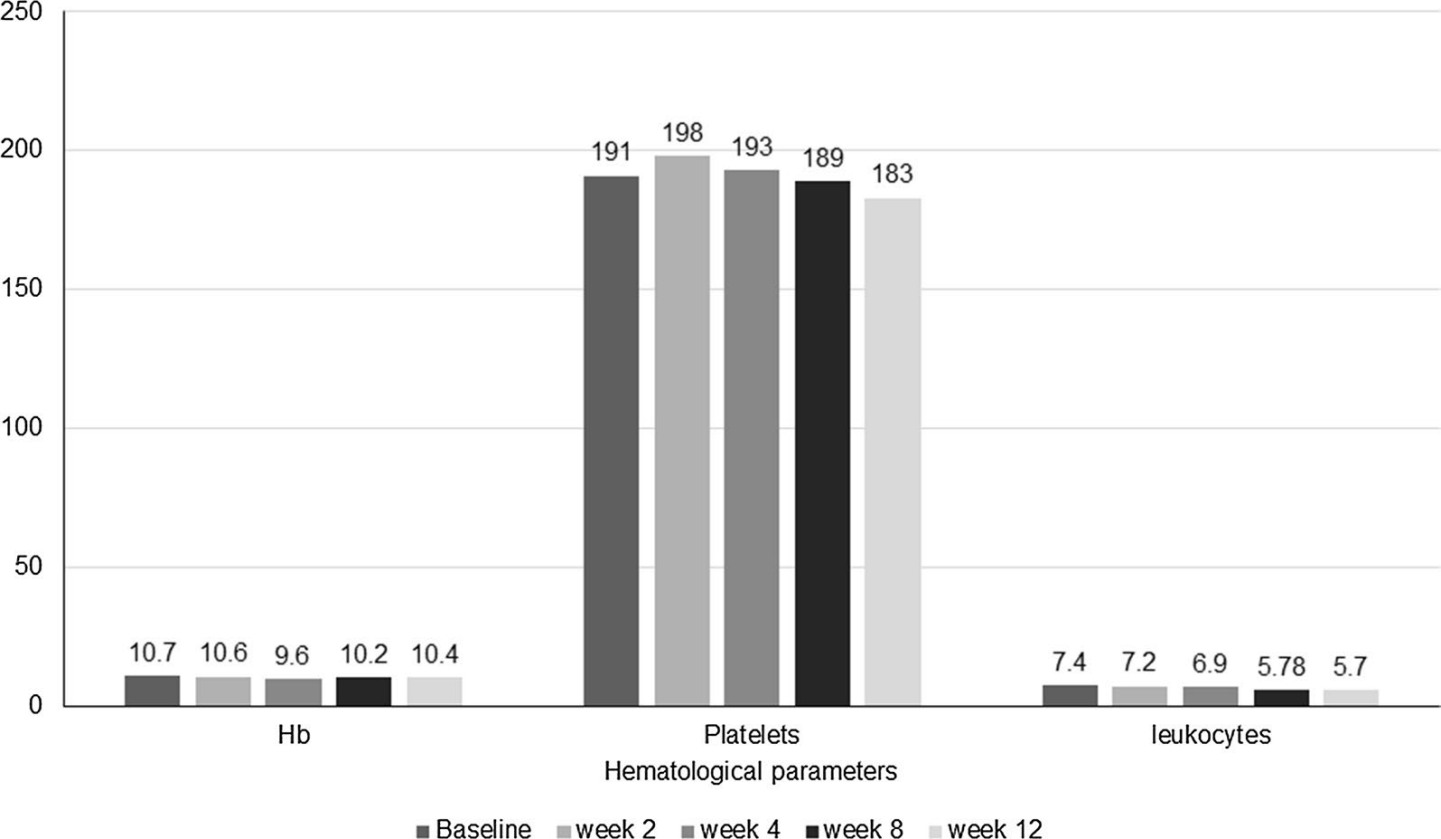
Median haematological parameters after [^177^Lu]Lu-DOTA-ZOL therapy

The laboratory parameters were tested and analysed for toxicity post-treatment (Table 6). Haematological serious adverse events (SAE) [grade III/IV toxicity] were not observed in any patient despite completing two cycles of [^177^Lu]Lu-DOTA-ZOL treatment. Though the difference in the haemoglobin levels post-second cycle was significant (*P* < 0.0001), only two patients in the series experienced grade II anaemia after the [^177^Lu]Lu-DOTA-ZOL therapy; nadir was around 4 weeks and subsequently recovered (Fig. 6). None of the patients had shown renal toxicity and other side effects like hypercalcemia.

**Table 6:** Laboratory parameters at baseline and post-[^177^Lu]Lu-DOTA-ZOL treatment

Parameters	Patients receiving single cycle			Patients receiving 2 cycles		
	Baseline (median, IQR)	Post-treatment (median, IQR)	*P* value	Baseline (median, IQR)	Post-treatment (median, IQR)	*P* value
Haemoglobin (g/dL)	10.2 (9.6–10.6)	10 (8.9–11)	0.129	14.7 (10–12.4)	13 (9.2–11)	< 0.0001
Platelets (lakhs/µL)	172 (140.2–198)	143 (129.2–198.5)	0.039	223 (164–284.25)	217 (170–275.5)	0.421
Leukocytes 10^9^/L	6760 (4210–11,800)	5400 (3700–10,800	0.291	6700 (4280–12,000)	5400 (3800–10,900)	0.298
Creatinine (mg/dL)	0.8 (0.63–0.96)	0.79 (0.6–0.94)	0.259	0.7 (0.7–0.9)	0.8 (0.7–0.8)	0.174
ALP (IU/L)	282 (190–480)	189 (140–280)	0.0001	278 (186–390)	230 (142–340)	0.003

*ALP* alkaline phosphatase, *IQR* interquartile range

## Discussion

Three independent randomised, placebo-controlled trials on zoledronic acid in about 3000 patients demonstrating hormone-resistant metastatic prostate cancer [33], breast cancer [34], have demonstrated a reduction in skeletal-related events (SREs) and proved it clinically effective. Subsequently, zoledronate received regulatory approval for the prevention of SREs and treating bone metastases. The labelling of zoledronate to beta- and alpha-emitting radionuclides like ^177^Lu and ^225^Ac, respectively, was a logical development [19, 20].

Though EDTMP has been proved effective for bone pain palliation [8], [^177^Lu]Lu-DOTA-ZOL proves superior to the former agent in certain aspects. In comparison with EDTMP, zoledronate demonstrates higher osteoclastic bone affinity and excellent anti-resorptive properties. While both the agents are non-bio-transformative, rapid clearance of [^177^Lu]Lu-DOTA-ZOL from blood, in contrast to [^177^Lu]Lu-EDTMP, dosimetric studies report a higher absorbed dose to the trabecular bone surface (12.17 vs. 10.02 mSv/MBq), cortical bone surface (9.524 ± 0.803 vs. 7.839 ± 0.655), and a noticeably lesser muscle uptake [27]. Therefore, [^177^Lu]Lu-DOTA-ZOL theoretically would have a better treatment efficacy. Moreover, unlike EDTMP, DOTA-ZOL complexes with ^68^ Ga and ^177^Lu/^225^Ac facilitate [19] a theranostic pair for practicing precision oncology.

However, to date, there is no study published in the literature showing therapeutic efficacy, safety, and toxicity of [^177^Lu]Lu-DOTA-ZOL in bone pain palliation in metastatic cancer patients. Hence, the current research focuses on the efficacy and safety study of [^177^Lu]Lu-DOTA-ZOL pain palliation therapy in patients with bone metastases.

Well, in line with the literature [1], 85% of the patients treated comprised of skeletal metastases from breast and prostate cancer. Interestingly, a significant component of our patients (70%) managed had an ECOG performance state of 3 or 4, and 35% of patients presented with either diffuse or super-scan reflecting the high tumour burden and poor ECOG status, which is a real-life scenario at the clinics. These sets of patients were refractory to the ongoing therapy options, including strong analgesics.

Khawar et al. [21] reported an estimated maximum tolerable dose (MTD) of [^177^Lu]Lu-DOTA-ZOL to be 3.6–5.0 GBq can be administered based on the 2 Gy bone marrow limit. In the present study, we administered a mean cumulative dose of 2.1 ± 0.6 GBq and ranged 1.3–2.7 GBq over a median of 2 cycles at three-monthly intervals; these administered activities were well within the prescribed limits. According to Khawar et al. [21], if the administered activities do not exceed MTD, the threshold adsorbed doses for the critical organ, namely kidneys, shall be within the predicted limit. Patients who responded from the first cycle of [^177^Lu]Lu-DOTA-ZOL were counselled or self-consented for the second cycle, while patients who were not willing to undergo the next cycle of treatment or those who did not experience any relief in pain after the first cycle of [^177^Lu]Lu-DOTA-ZOL were not treated further. Another interesting find observed was that those patients who experienced minimal response from the 1st cycle of [^177^Lu]Lu-DOTA-ZOL treatment when re-challenged with the 2nd cycle did not encounter further reduction in the pain.

The current study reports an ORR of 90%, which is well within the range of response rate with other bone-seeking pain palliating agent [^177^Lu]Lu-EDTMP ranging between 83 and 86% [8, 9]. Interestingly, pain relief was initiated within ≤ 7 days after the [^177^Lu]Lu-DOTA-ZOL treatment and lasted up to 10 months. In a previous study using [^177^Lu]Lu-EDTMP, we had reported response durations that varied from 2 weeks to 4 months from the onset of pain relief [8]. Detailed analysis revealed a similar ORR of 82% in prostate cancer patients when compared to the historic [^177^Lu]Lu-EDTMP study (ORR 84%) [8]. However, a remarkably high ORR of 100% was observed in the breast cancer cohort of the present study in contrast to 92% in the historic [^177^Lu]Lu-EDTMP study [8]. Though data regarding the response pattern in bone metastases from lung cancer are limited, Ye et al. [35] reported a treatment efficacy rate of 75.4% in the lung cancer group, but with 2.22 MBq/kg ^89^SrCl_2_. In agreement with our results, they reported efficacy was lower in patients with bone metastases from the lung cancer sub-group than in those with bone metastasis from breast and prostate cancer [35].

The 1-year survival rate was 55.4% and was significantly higher when compared to that of the [^177^Lu]Lu-EDTMP historical data set [35% and 38% in the two-level dose group] [8], but drastically dropped to 26% at 13 months. This drop of overall survival is not clearly understood; however, it could be attributed to the aggressive biology of the tumour and the extent of spread. Among the cancer types, similar overall survival was noted in patients with breast and prostate cancer (13 months) and a slightly decreased survival in bone metastases patients with primary lung cancer. Attributed to the superior radiobiological properties of the alpha emitter, radium-223 chloride, Parker et al. [4] in their RCT, involving 921 patients with bone metastases from prostate cancer, outlined an OS of 14.9 months. Nilsson et al. [36] performed a survival follow-up of the phase-II RCT in which bone metastases patients from prostate cancer were treated with radium-223 chloride, and their study findings suggested a survival benefit of 65 weeks versus 46 weeks in patients treated with a single dose of radium-223 chloride and placebo group, respectively.

Analgesic consumption was recorded according to the analgesic scoring of EORTC protocol. Among our recruited patients, 15 patients were on opioid analgesic morphine before radionuclide therapy out of whom 12 responded to [^177^Lu]Lu-DOTA-ZOL treatment and thus were weaned off morphine. It is these set of patients whom the pain palliation was most beneficial as they have exhausted the available pain relief options. Interestingly, reassuring results were obtained with [^177^Lu]Lu-DOTA-ZOL in patients demonstrating extensive skeletal metastases and those presenting with superscan or diffuse involvement of the bone. The relief in pain was also reflected in their KPS and ECOG performance status. Toxicity related to [^177^Lu]Lu-DOTA-ZOL therapy was minimal, and no grade III/IV toxicities were documents.

## Limitations

The significant limitations of this cohort study are the non-randomised study, and no parallel arm was used. Although the results of [^177^Lu]Lu-DOTA-ZOL were comparable to historical data of [^177^Lu]Lu-EDTMP, a randomised control trial to compare the efficacy and safety of two bone-seeking palliative agents is urgently desired to take this agent further.

## Conclusion

[^177^Lu]Lu-DOTA-ZOL is safe, effective, and an ideal agent in the treatment of metastatic bone pain. Thanks to its characteristics as theranostics, it allows for patient-individual therapies and perfectly matches the expectations for precision oncology. Interestingly, [^177^Lu]Lu-DOTA-ZOL is not only effective in addressing bone metastases derived from breast cancer; it shows clear evidence also for prostate and lung cancers. [^177^Lu]Lu-DOTA-ZOL pain palliation treatment demonstrated an ORR of 90% with 27.5% of complete response and 50% of partial response.

## Data Availability

Data and material are available.

## Abbreviations

IQR: Interquartile range
VAS: Visual analogue score
AS: Analgesic score
ECOG: Eastern Cooperative Oncology Group Assessment performance status
KPS: Karnofsky performance status
SREs: Skeletal-related events
EBRT: External beam radiotherapy
EDTMP: Ethylene diamine tetramethylene phosphonic acid
BPAMD: 4-{[(Bis(phosphonomethyl)) carbamoyl]methyl}-7,10-bi (carboxymethyl)-1,4,7,10-tetraazacyclododec-1-Yl) acetic acid
DOTA-ZOL: Zoledronate
BSA: Body surface area
EORTC: European Organization of Research and Treatment of Cancer
LuCl_3-_: Lutetium chloride
GFR: Glomerular filtration rate
CBC: Complete blood counts
KFT: Kidney function tests
ALP: Alkaline phosphatase
CR: Complete remission
PR: Partial remission
MR: Minimal response
NR: No response
PET/CT: Positron emission tomography/computed tomography
NCI-CTC: National Cancer Institute’s Common Toxicity Criteria
OS: Overall survival
SD: Standard deviation
MTD: Maximum tolerable dose
ORR: Overall response rate
